# Improved survival in several cancers with use of H_1_-antihistamines desloratadine and loratadine

**DOI:** 10.1016/j.tranon.2021.101029

**Published:** 2021-02-05

**Authors:** Ildikó Fritz, Philippe Wagner, Håkan Olsson

**Affiliations:** a Department of Cancer Epidemiology, Clinical Sciences, Lund University, Lund, Sweden; b Department of Oncology and Pathology, Clinical Sciences, Lund University, Lund, Sweden

**Keywords:** Epidemiology, Histamine H1 antagonists, Histamine antagonists, Chemotherapy, Immunotherapy

## Abstract

**Background:**

We have previously shown an association with substantially improved survival in breast cancer and melanoma for desloratadine and loratadine users, and set out to find whether an improved survival can be seen in tumors with and without a known response to immune checkpoint therapy, such as anti-CTLA-4 or anti-PD-1.

**Methods:**

We investigated survival and use of six common H_1_-antihistamines (cetirizine, clemastine, desloratadine, ebastine, fexofenadine and loratadine) in a nation-wide cohort of all 429,198 Swedish patients with ten types of immunogenic (gastric, colorectal/anal, pancreatic, lung, breast, prostate, kidney, and bladder cancer, melanoma and Hodgkin lymphoma) and six non-immunogenic (liver, uterine, ovarian, brain/CNS, and thyroid cancer and non-Hodgkin lymphoma) tumors diagnosed 2006–2017. Follow-up was until 2019–02–24.

**Findings:**

Desloratadine use was associated with an improved survival for all immunogenic tumors, but not for the non-immunogenic ones. Loratadine use was associated with improved survival for some tumors. Use of the other antihistamines could not be shown to be consistently associated with improved survival to a statistically significant degree.

**Interpretation:**

Our hypothesis is that our findings result from immune checkpoint inhibition, and we believe both desloratadine and loratadine should be tested in randomized clinical trials as treatment of immunogenic tumors, with priority given to trials of desloratadine as treatment of tumors with few therapy options and dismal prognoses, such as pancreatic cancer. If our results can be confirmed in a clinical setting, new, potentially curative, therapies could result for several tumors, including ones with dire prognoses and limited treatment options.

## Highlights

• Improved cancer survival with use of antihistamines desloratadine and loratadine.
• Improved survival seen in tumors that respond to immune checkpoint therapy.
• A – potentially immunological – anti-tumor effect of desloratadine and loratadine.

## Introduction

Cancer therapy can be severely limited depending on tumor type or subtype, and there is always a need for new and improved anti-cancer drugs, especially for malignancies with dismal prognoses like pancreatic cancer [1]. Repurposing of existing medication is a way to meet that need in a both time- and cost-effective manner [2,3]. Antihistamines targeting histamine receptor H_1_ make excellent candidates for drug repurposing for cancer therapy: they are safe drugs with minimal side effects that are well tolerated by most people, and evidence that they may be effective against several tumors is mounting [4], [5], [6], [7], [8], [9], [10], [11], [12], [13], [14], [15], [16], [17], [18]. Different mechanisms have been proposed for this potential effect: most are thought to be either wholly or partly histamine receptor H_1_ independent [9,[13], [14], [15],^17^], either involving lysosomal cell death [9,19,20] or immunological pathways [21,22]. Some antihistamines, like desloratadine, fexofenadine and loratadine, have been shown to have anti-inflammatory effects [23], that are thought to depend on their strong inverse histamine agonism, inhibiting even the basal signaling of the histamine receptor H_1_ [23], [24], [25]. Desloratadine can also stabilize mast cell membranes, and thereby prevent the release of histamine [10,26,27]. Histamine promotes the immunoregulatory activity of myeloid-derived suppressor cells and the Th2-skewing immune response [21,22,28,29]. The Th1-response has been shown to be important for survival in colorectal cancer [28]. We have previously found that use of the H_1_-antihistamines desloratadine and loratadine is associated with substantially improved survival in both breast cancer [30] and cutaneous malignant melanoma [31], and in this meta-analysis of our data, we investigate whether a similar association can be seen across multiple tumor types, with and without a known response to treatment with immune checkpoint inhibitors, such as anti-CTLA-4 or anti-PD-1, to shed further light on the possible anti-tumor effect of these antihistamines, and whether it may be immunological in nature.

## Methods

### Registers and data handling

In this meta-analysis, we investigated antihistamine use and survival in 16 different tumor types, divided into two groups based on whether they have any known response to immune checkpoint therapy (hereafter referred to as immunogenic vs non-immunogenic tumors). Ten tumor types were included in the immunogenic group: gastric cancer, colorectal and anal cancer, pancreatic cancer, lung cancer, breast cancer, prostate cancer, kidney cancer, bladder cancer, melanoma and Hodgkin lymphoma. The non-immunogenic group included six tumor types: liver and biliary tract cancer, uterine cancer, ovarian cancer, brain and CNS tumors, thyroid cancer, and non-Hodgkin lymphoma. Our study populations include all 429,198 cases of newly diagnosed tumors of these cancer types in the entire Swedish population between 2006 and 2017 included in the Swedish Cancer Register (SCR), a database of all cancer cases in Sweden since 1958. Antihistamine use (defined as use of the six most common H_1_-antihistamines used among Swedish cancer patients, namely cetirizine, clemastine, desloratadine, ebastine, fexofenadine and loratadine) was established using the Swedish Prescribed Drug Register, a record of all dispensed prescribed pharmaceuticals in Sweden since July 1st, 2005. Prescription-free use, non-dispensed doses, or use of other, uncommon, antihistamines is here considered non-use. Follow-up was until February 24th, 2019. Causes of death were obtained from the Swedish Cause of Death Register, a record of all deaths since 1952. Data was pseudo-anonymized (with a key kept for a limited time by The National Board of Health and Welfare in Sweden), and the study was approved by the Regional Ethics Board.

### Statistical analyses

Peri-diagnostic antihistamine use in relation to tumor-specific survival was analyzed separately for each tumor type (and together for the immunogenic tumors and non-immunogenic tumors respectively). Peri-diagnostic use was defined as the main antihistamine (in terms of dispensed defined daily doses, or DDDs) used within six months pre-diagnosis and six months post-diagnosis. Crude time-specific survival in relation to peri‑diagnostic desloratadine and loratadine use was presented in Kaplan-Meier plots for each tumor type. Subsequent analyses of use of all six antihistamines were done using Cox regression models with time to tumor-specific death, or censoring due to migration or study end, in years starting from six months after the time of diagnosis, as time scale. Analyses were stratified for patient gender (as a proxy for sex) and age at diagnosis, which was divided into ten-year age categories. Results were presented in a forest plot and summarized using fixed effects meta-analysis by means of the R package meta [32]. All analyses were done using R [33]. P-values less than 0.05 were considered statistically significant.

## Results

Out of the 429,198 cases of cancer in our total study populations, the most common cancer was prostate cancer (*n* = 111,664), followed by breast cancer (*n* = 76,395) and colorectal cancer (*n* = 60,910). The most common antihistamine used was cetirizine with 8,606 users, followed by desloratadine (8,269 users), clemastine (8,167 users) and loratadine (5,957 users). Ebastine and fexofenadine had much fewer users. 396,667 individuals did not use any of the six H_1_-antihistamines and were thus classified as non-users. 77,900 deaths occurred during our study period, most deaths occurred among the lung cancer patients (16,249 deaths), followed by colorectal cancer (14,599 deaths) and prostate cancer (11,025 deaths). (Supplemental Table 1.)

**Fig. 1:**
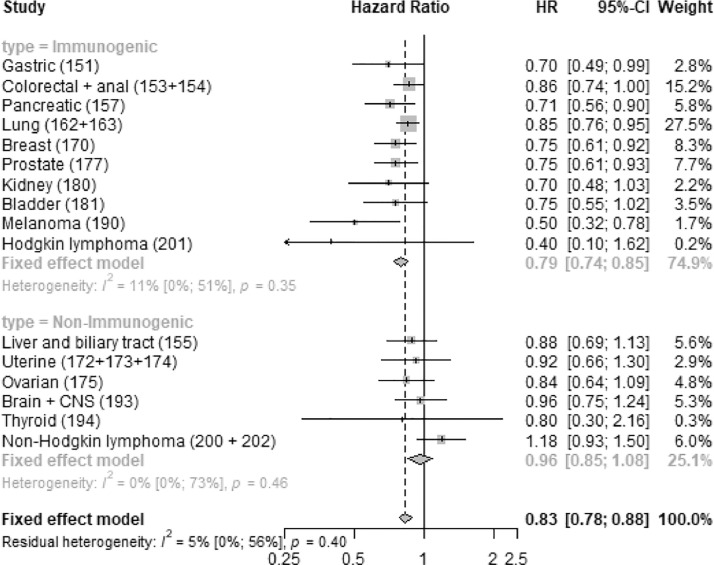
**Desloratadine use and tumor-specific mortality.** Forest plot showing hazard rate ratios associated with peri‑diagnostic desloratadine use for each tumor type, as well as for the immunogenic and non-immunogenic groups pooled, together with measures of heterogeneity for the groups.

We found that desloratadine use was associated with an improved tumor-specific survival for all the immunogenic tumors but none of the non-immunogenic ones (Fig. 1).

**Fig. 2:**
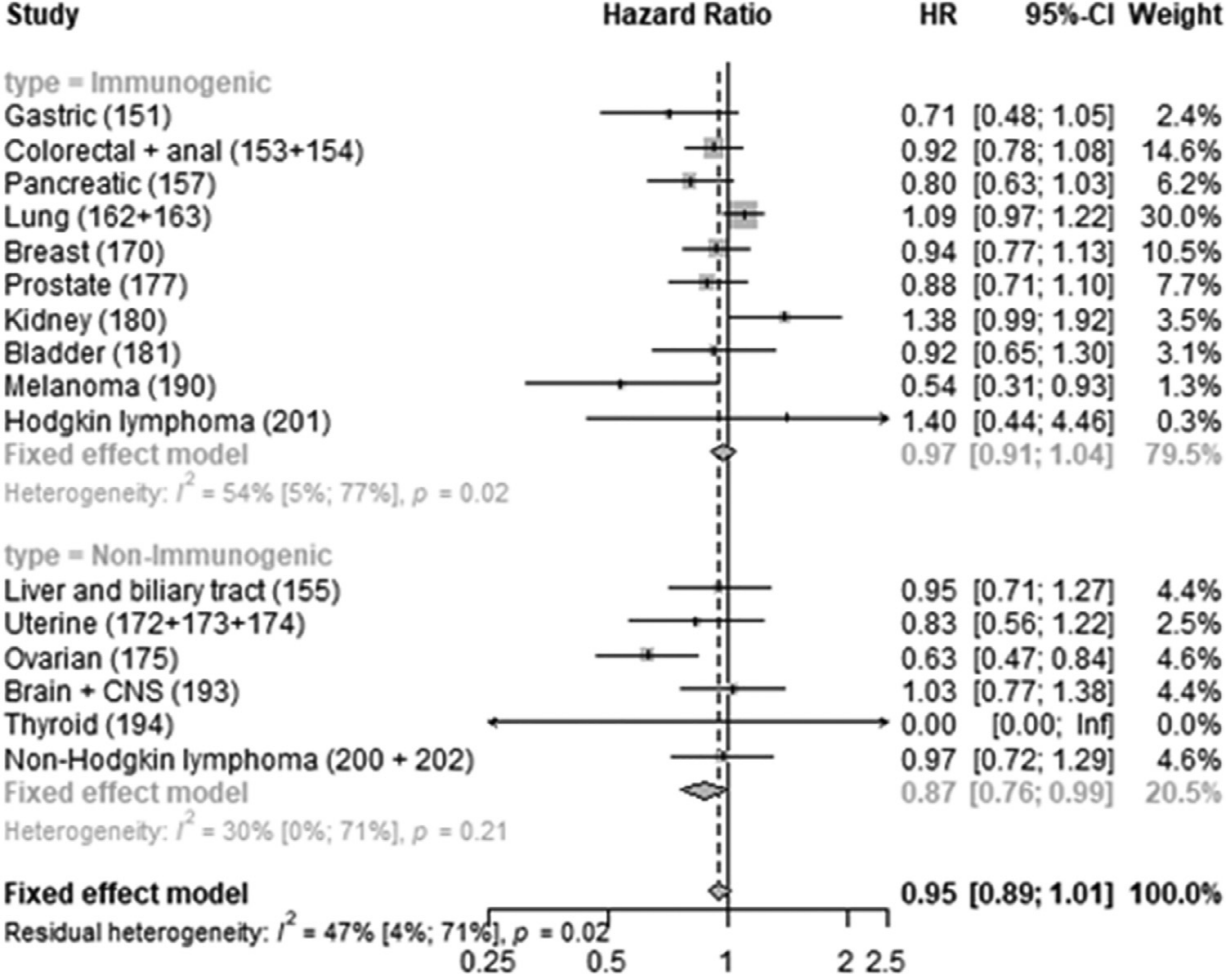
**Loratadine use and tumor-specific mortality.** Forest plot showing hazard rate ratios associated with peri‑diagnostic loratadine use for each tumor type, as well as for the immunogenic and non-immunogenic groups pooled, together with measures of heterogeneity for the groups.

Loratadine use was also associated with improved tumor-specific survival for some of the immunogenic tumors, especially melanoma, but confidence intervals were wider than for desloratadine. Loratadine use was also associated with improved tumor-specific survival for ovarian cancer. (Fig. 2.)

Cetirizine use was associated with improved survival in gastric, pancreatic and ovarian cancer (Supplemental Fig. 1). We saw no statistically significant association with improved survival for any of the other antihistamines (Supplemental Figs. 2–4). The tumor-specific mortality for desloratadine users relative to non-users with immunogenic tumors when plotted as survival functions show parallel functions after approximately 2–4 years (Fig. 3, Fig. 4, Fig. 5, Fig. 6 and Supplemental Figures 5–10).

**Fig. 3:**
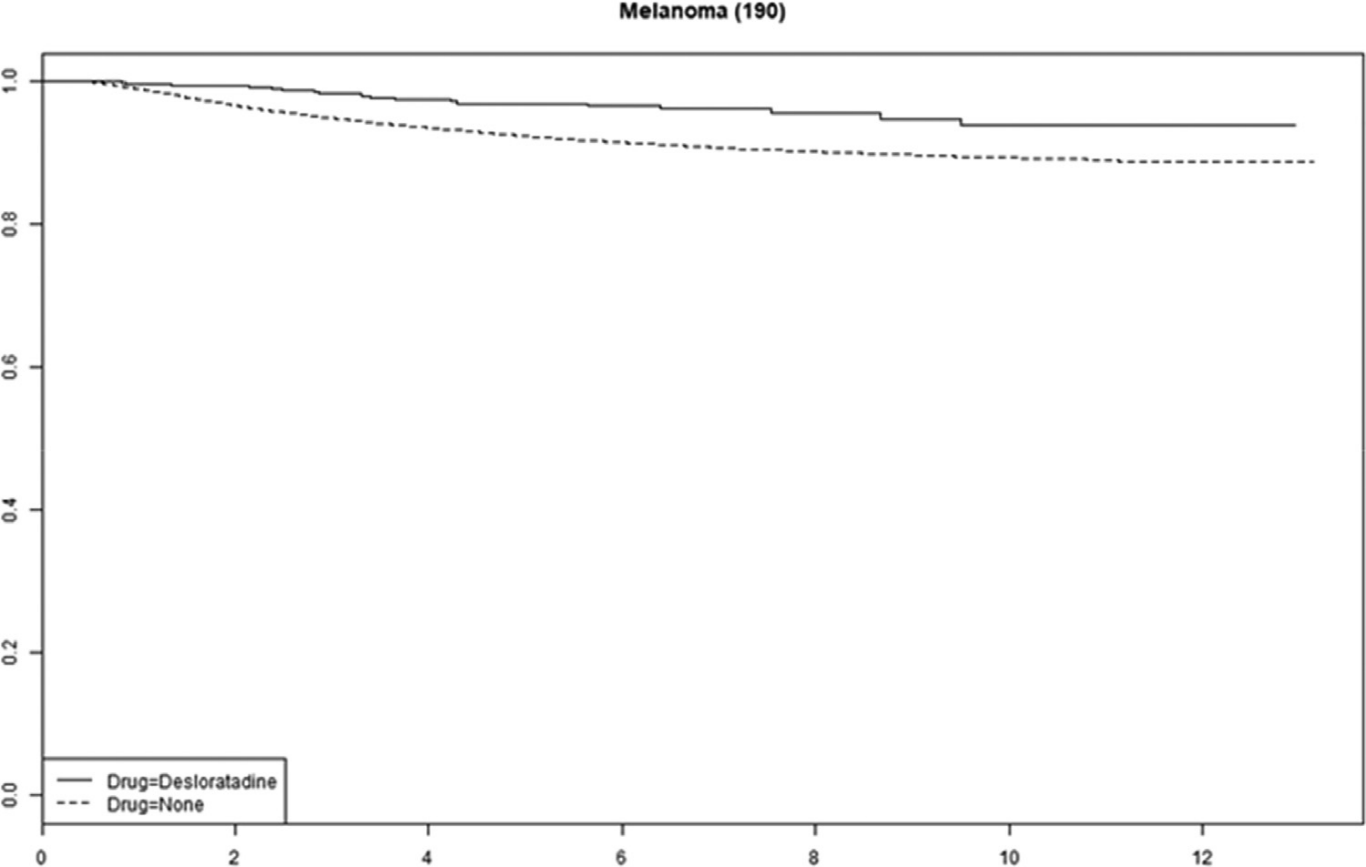
**Tumor-specific survival of desloratadine users vs non-users with melanoma.** Melanoma-specific survival probability plotted against time since diagnosis in years.

**Fig. 4:**
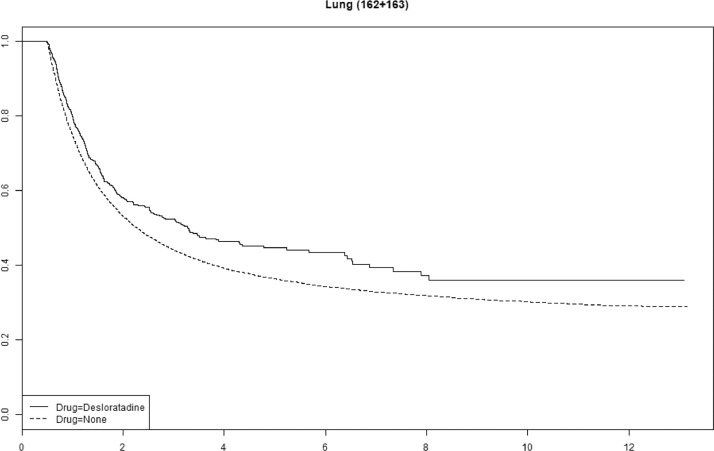
**Tumor-specific mortality of desloratadine users vs non-users with lung cancer.** Lung cancer-specific survival probability plotted against time since diagnosis in years.

**Fig. 5:**
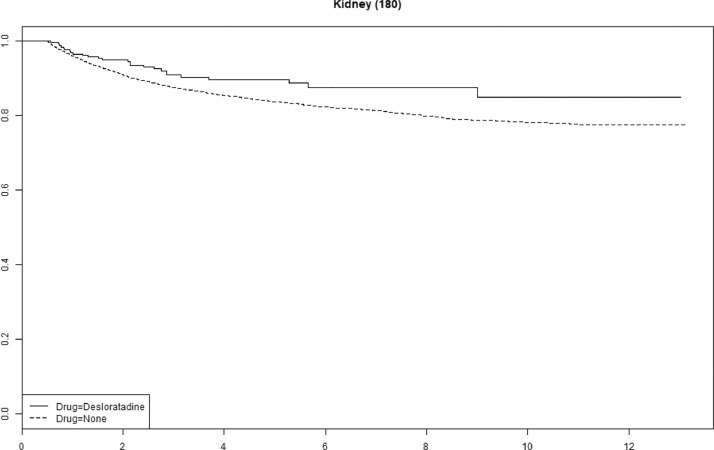
**Tumor-specific mortality of desloratadine users vs non-users with kidney cancer.** Kidney cancer-specific survival probability plotted against time since diagnosis in years.

**Fig. 6:**
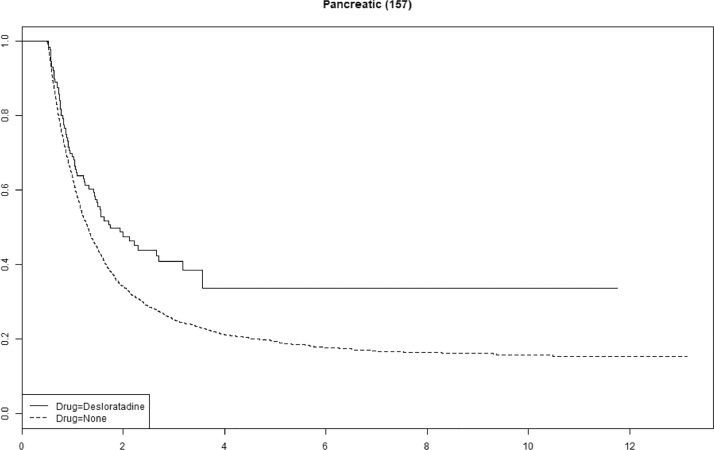
**Tumor-specific mortality of desloratadine users vs non-users with pancreatic cancer.** Pancreatic cancer-specific survival probability plotted against time since diagnosis in years.

## Discussion

Our hypothesis is that our findings in this meta-analysis, as well as in our previous work on breast cancer and melanoma, have to do with immune checkpoint inhibition, though others have presented evidence which suggests that the potential effect may depend on lysosomal cell death [9,19,20]. It remains to be seen what the pathways involved are, however, as these drugs have cleared all toxicity screens and are currently in use globally, they make excellent candidates for randomized clinical trials.

The immunogenic nature of different tumors is not always a completely straightforward issue. For some tumor types, most subtypes may be immunogenic or non-immunogenic in nature, while for others, subtypes may vary, and thus to classify correctly is impossible unless all tumor subtypes are known. We have used known response to immune checkpoint therapy as a proxy for immunogenicity for our groupings. There is ample evidence of response to immune checkpoint therapy for melanoma, Hodgkin lymphoma and bladder, breast, colorectal, gastric, kidney and lung cancer [34], [35], [36], [37], however, while there are indications and results that support the inclusion of pancreatic and prostate cancer in this group [38], [39], [40], [41], the basis for their inclusion is significantly weaker. We have nevertheless chosen to classify these tumors as immunogenic, based on the available evidence, and we have chosen to present our results not only for the groups but also for each tumor type. Furthermore, it could be argued that ovarian cancer should also be included in the immunogenic group based on *in vitro* and *in vivo* studies, however, as clinical trials have failed to show the same response to immunotherapies [42], we have chosen to here classify ovarian cancer as non-immunogenic. It remains to be seen if and how immunotherapy can successfully be implemented in ovarian cancer treatment, and whether any antihistamines could play a role here too, as we saw an association with improved ovarian cancer survival with both cetirizine and loratadine use. That the heterogeneity measure for desloratadine is low supports our groupings.

In our previous studies on breast cancer and cutaneous malignant melanoma, we could adjust for a number of factors, especially in the melanoma study where a quality register was used, and we showed that the potential effect was present in both the crude and adjusted analyses [30, 31]. Here, we had no such quality register data available to us, but even without these adjustments, similar associations can be seen.

A possible bias in this kind of study may arise when there are unmeasured risk factors affecting both the outcome and cancer incidence, in which case cancer may become a collider if the exposure also influences cancer incidence. However, a study on cancer risk among allergy patients by Hemminki et al. found not only that there was no overall increased risk of cancer for allergy patients, but also that allergy patients had a lower risk of some cancers and a higher risk for some cancers [43] and other studies on cancer risk for patients with allergy/atopy have shown both reduced and increased and no difference in cancer risk for atopic patients [44], [45], [46], [47], indication that there may not be such a systematic bias present. We also used controls from another study and checked whether there was an increased risk of cancer for desloratadine users, and found no increased risk (data not shown), although we did not have enough events to exclude this as the reason for not finding any increased risk. While this is not conclusive proof that bias of this kind is not an issue here, these points, together with the effects seen of antihistamines on cancer cells in previous studies, nevertheless suggest that this study may not need to suffer from any such systematic bias. Another issue that may be present in this kind of study is confounding by indication, however, we do not believe that to be an issue here, most importantly because there is not a single common indication for all antihistamine users in this study. In addition, there is no overall association between altered prognosis or survival for those with allergic conditions [47], [48], [49], [50], [51], [52], [53], [54], [55], suggesting that while the heightened immunosurveillance in those with allergic conditions may sometimes result in a decreased cancer risk [47], it does not confer any survival benefit to those that do develop tumors [44], and use of different antihistamines is not associated with uniform cancer survival [9,18,56].

While three antihistamines have been available without a prescription in Sweden throughout the study period (cetirizine, loratadine and ebastine), precluding us from being able to appreciate their full exposure in our population, fexofenadine was not made available without a prescription until 2011 in Sweden, and desloratadine until 2014, while clemastine is not available prescription-free, thus a full or nearly full exposure can be appreciated for those three antihistamines. We therefore expect that the potential effect of loratadine may in reality be greater than what we can show here, based on a dilution due to a background exposure in the population. The current prescription-free availability of desloratadine in Sweden also means that this study cannot be replicated in the Swedish population, or elsewhere where these H_1_-antihistamines are available without a prescription.

Furthermore, as immunotherapy is now an integral part of melanoma treatment, as well as the treatment of several other tumors, but was not used for the treatment of melanoma during most of the study period, we can here evaluate the potential effect of desloratadine and loratadine without any confounding due to effects of immunotherapy. Our hypothesis is that if these antihistamines are given together with modern or forthcoming immunotherapeutic agents, the effects could be synergistic or additive and enhance one another. Important to note is also that for this reason, any studies or trials of novel immunotherapies in cancer treatment should take into account this potential antihistamine effect, as antihistamines are often given to alleviate side effects of these therapies, which may introduce confounding into the analyses of such trials and studies.

As desloratadine is the active metabolite of loratadine, it is possible that at least part of the potential loratadine effect is in actuality a desloratadine effect. Alternatively, a heterogeneity among tumor types in regard to this potential effect may explain the differences seen.

The parallel survival functions between users and non-users of desloratadine after two to four years suggest that a curative effect may be present for approximately 10% of patients in some tumor types, which is similar to the proportion of patients who respond to immune checkpoint therapy, however, these analyses are crude, and after proper adjustments estimates may change.

As this study cannot readily be reproduced, due to the window of prescription-only use of desloratadine and the introduction of immunotherapy for melanoma among other tumors, we recommend that clinical trials should be initiated, alongside further studies into the mechanism behind this potential effect. While we propose desloratadine and loratadine as novel anti-cancer drugs, they are not novel drugs, and are in fact routinely given to cancer patients and others with serious disease with few or no adverse effects.

We believe that there is already a compelling case for further studies of H_1_-antihistamines as potential cancer treatment, as well as for initiation of clinical trials of mainly desloratadine and loratadine, as treatment of both breast cancer and cutaneous malignant melanoma. Based on what we present here, that pool should be extended to include at least all other immunogenic tumors in our study and priority should be given to trials of desloratadine as treatment of tumor types with dismal prognoses. The potential gain in prognosis, with treatments with little or no toxicity, could be substantial, especially for patients who currently have few treatment options.

## Financial support

This research was funded in part by grants from the Swedish Cancer Society, Henning & Ida Persson's research foundation, Berta Kamprad's foundation, and local hospital foundations awarded to Håkan Olsson.

## CRediT authorship contribution statement

**Ildikó Fritz:** Conceptualization, Investigation, Writing - original draft, Writing - review & editing, Visualization, Project administration. **Philippe Wagner:** Methodology, Software, Validation, Formal analysis, Data curation, Writing - review & editing, Visualization. **Håkan Olsson:** Conceptualization, Writing - review & editing, Supervision, Project administration, Funding acquisition.

## Conflict of Interest disclosure statement

The authors report no conflicts of interest, however, Håkan Olsson holds patents for desloratadine in the treatment of breast cancer in the US, EU and Japan through Belina AB, and has patents pending for desloratadine in other immunogenic cancer therapy through HAKMED AB.

## Appendix Supplementary materials

Image, application 1
