# Supplementary material for: Improved survival in several cancers with use of H1-antihistamines desloratadine and loratadine
Source: Transl Oncol. 2021 Feb 5;14(4):101029. doi: 10.1016/j.tranon.2021.101029 (PMC7868613; doi:10.1016/j.tranon.2021.101029)
Supplement: Supplementary file 1 [file mmc1.docx]

**SUPPLEMENTAL TABLE**

**Supplemental Table 1. Study population characteristics.**

| Tumor type | ICD-7 | N | Tumor-specific deaths (n) | | Median follow-up (years) | | Mean age (years) | | Men (n) | Women (n) | | Non-users (n) | | Clemastine users (n) | | Cetirizine users (n) | | Loratadine users (n) | | Ebastine users (n) | | Fexofenadine users (n) | | Desloratadine users (n) | |  |
| --- | --- | --- | --- | --- | --- | --- | --- | --- | --- | --- | --- | --- | --- | --- | --- | --- | --- | --- | --- | --- | --- | --- | --- | --- | --- | --- |
| ***Immunogenic*** | | | | | |  | |  | | |  | |  | |  | |  | |  | |  | |  | |  | |
| Gastric | 151 | 5982 | 2704 | | 1∙9 | | 69∙7 | | 3686 | 2296 | | 5593 | | 120 | | 89 | | 75 | | 9 | | 4 | | 92 | |  |
| Colorectal +anal | 153 +154 | 60910 | 14599 | | 4∙1 | | 70∙9 | | 31642 | 29268 | | 57144 | | 1105 | | 852 | | 702 | | 125 | | 49 | | 933 | |  |
| Pancreatic | 157 | 6001 | 4249 | | 1∙2 | | 68∙2 | | 2896 | 3105 | | 4746 | | 886 | | 119 | | 98 | | 15 | | 7 | | 130 | |  |
| Lung | 162 +163 | 28541 | 16249 | | 1∙7 | | 69∙5 | | 13703 | 14838 | | 25948 | | 660 | | 636 | | 517 | | 77 | | 28 | | 675 | |  |
| Breast | 170 | 76395 | 6252 | | 5∙9 | | 64 | | 0 | 76395 | | 68798 | | 1265 | | 2409 | | 1623 | | 281 | | 127 | | 1892 | |  |
| Prostate | 177 | 111664 | 11025 | | 5∙4 | | 69∙8 | | 111664 | 0 | | 106783 | | 887 | | 1187 | | 972 | | 200 | | 96 | | 1539 | |  |
| Kidney | 180 | 12515 | 1997 | | 4∙4 | | 67∙4 | | 7789 | 4726 | | 11597 | | 230 | | 202 | | 172 | | 34 | | 18 | | 262 | |  |
| Bladder | 181 | 27142 | 3781 | | 4∙2 | | 72∙5 | | 20294 | 6848 | | 25674 | | 338 | | 358 | | 263 | | 50 | | 29 | | 430 | |  |
| Melanoma | 190 | 33810 | 2532 | | 5∙1 | | 63∙4 | | 16996 | 16814 | | 32017 | | 232 | | 430 | | 351 | | 73 | | 45 | | 662 | |  |
| Hodgkin lymphoma | 201 | 1839 | 158 | | 6∙0 | | 47∙5 | | 1027 | 812 | | 1526 | | 105 | | 77 | | 39 | | 6 | | 3 | | 83 | |  |
| ***Non-immunogenic*** | | | |  | |  | |  | | |  | |  | |  | |  | |  | |  | |  | |  | |
| Liver +biliary | 155 | 6027 | 3059 | | 1∙7 | | 68 | | 3513 | 2514 | | 5014 | | 631 | | 120 | | 91 | | 17 | | 5 | | 149 | |  |
| Uterine | 172 +173 +174 | 16184 | 2191 | | 5∙3 | | 69∙3 | | 0 | 16184 | | 14793 | | 330 | | 424 | | 250 | | 42 | | 23 | | 322 | |  |
| Ovarian | 175 | 7476 | 2790 | | 3∙6 | | 64 | | 0 | 7476 | | 6231 | | 447 | | 353 | | 215 | | 20 | | 9 | | 201 | |  |
| Brain +CNS | 193 | 12737 | 3075 | | 4∙5 | | 57∙8 | | 5709 | 7028 | | 11587 | | 328 | | 261 | | 203 | | 34 | | 17 | | 307 | |  |
| Thyroid | 194 | 4633 | 262 | | 5∙6 | | 54∙3 | | 1301 | 3332 | | 4242 | | 71 | | 82 | | 62 | | 19 | | 8 | | 149 | |  |
| Non-Hodgkin lymphoma | 200 +202 | 17342 | 2977 | | 4∙7 | | 67∙8 | | 9746 | 7596 | | 14974 | | 532 | | 1007 | | 324 | | 36 | | 26 | | 443 | |  |

**SUPPLEMENTAL FIGURES**

**
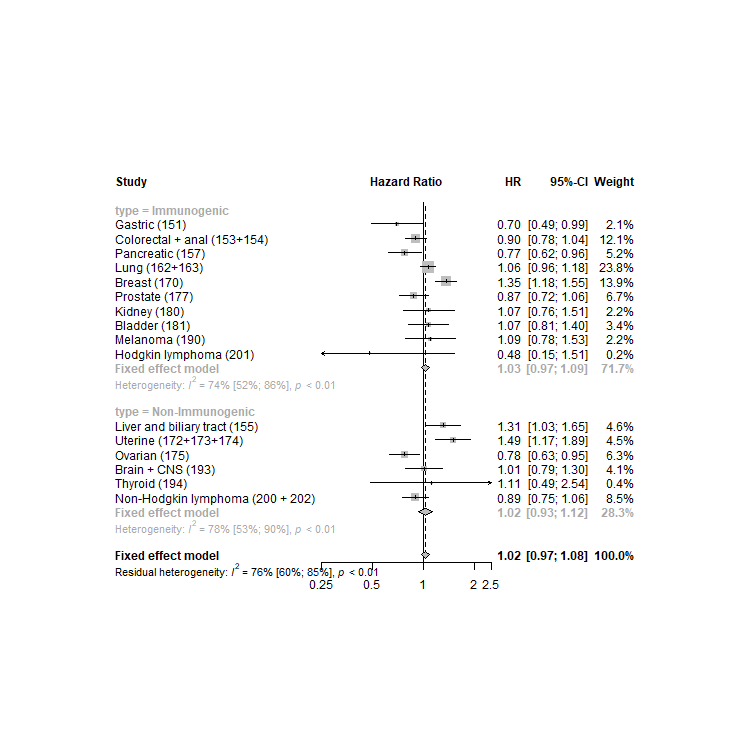
Supplemental Figure 1. Cetirizine use and tumor-specific mortality.** Forest plot showing hazard rate ratios associated with peri-diagnostic cetirizine use for each tumor type together with measures of heterogeneity for immunogenic and non-immunogenic subgroups, as well as for both groups pooled.

**
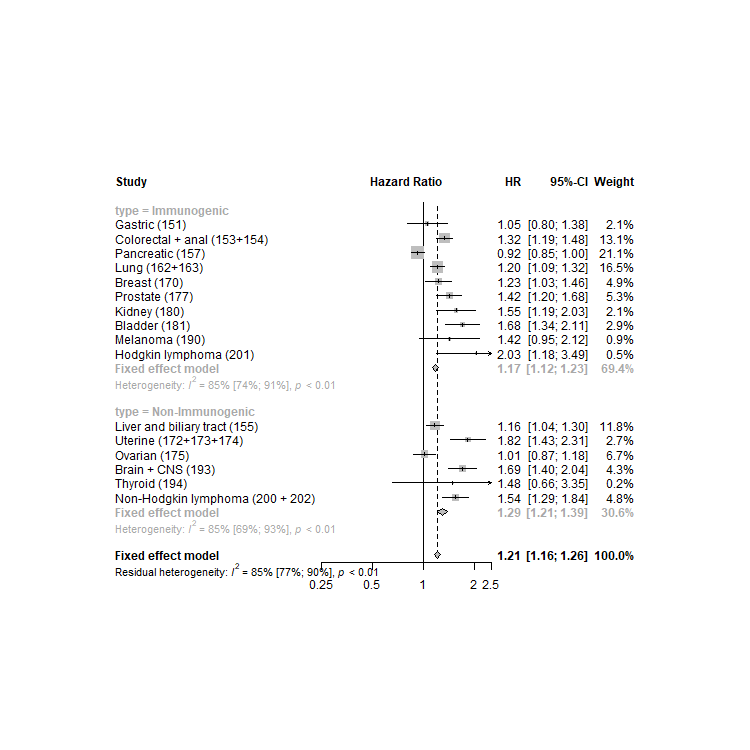
**

**Supplemental Figure 2. Clemastine use and tumor-specific mortality.** Forest plot showing hazard rate ratios associated with peri-diagnostic clemastine use for each tumor type together with measures of heterogeneity for immunogenic and non-immunogenic subgroups, as well as for both groups pooled.

**
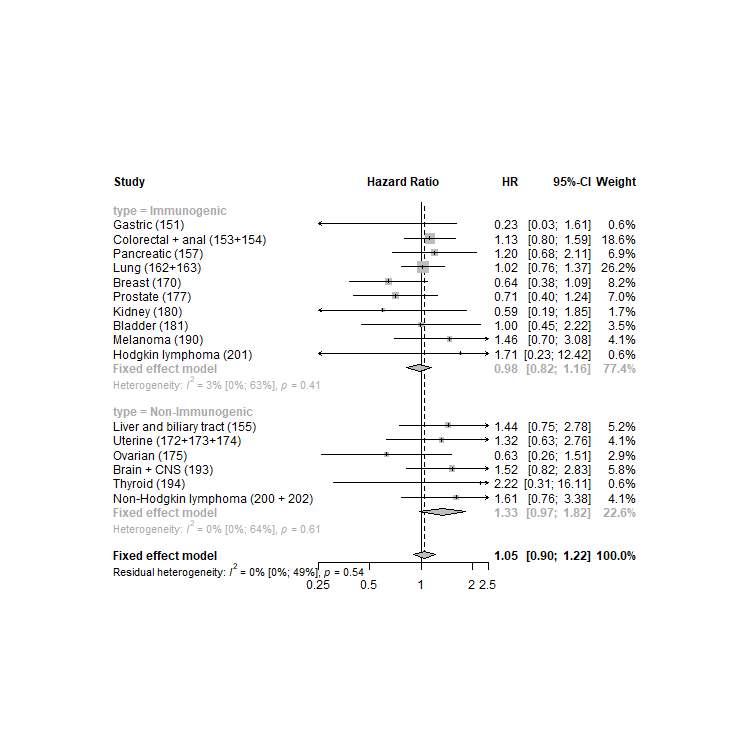
**

**Supplemental Figure 3. Ebastine use and tumor-specific mortality.** Forest plot showing hazard rate ratios associated with peri-diagnostic ebastine use for each tumor type together with measures of heterogeneity for immunogenic and non-immunogenic subgroups, as well as for both groups pooled.

**
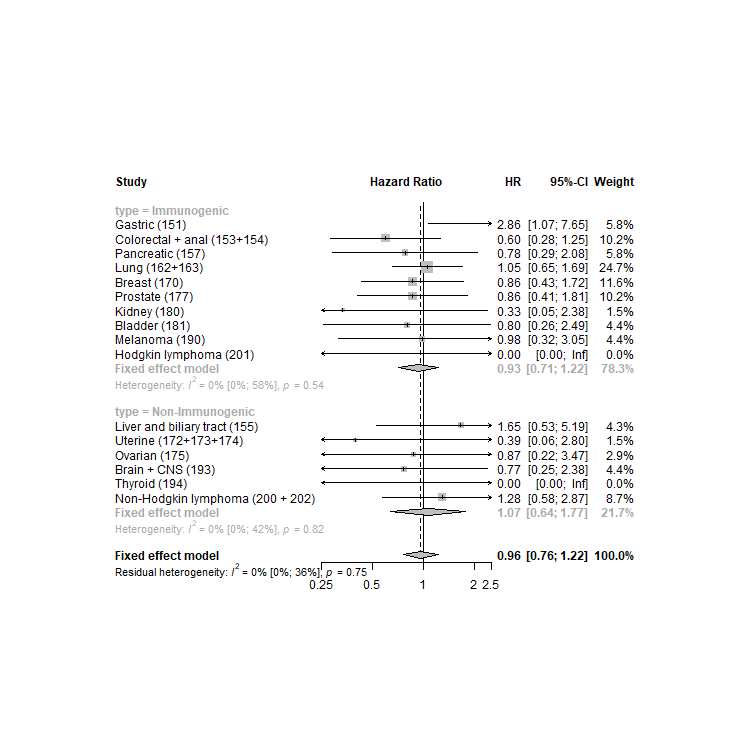
**

**Supplemental Figure 4. Fexofenadine use and tumor-specific mortality.** Forest plot showing hazard rate ratios associated with peri-diagnostic fexofenadine use for each tumor type together with measures of heterogeneity for immunogenic and non-immunogenic subgroups, as well as for both groups pooled.

**
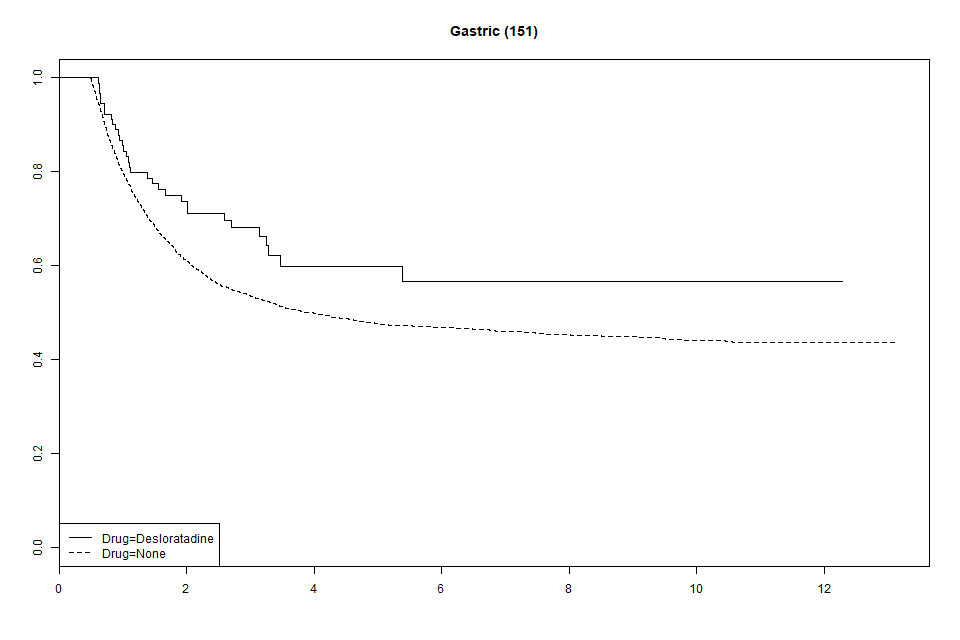
**

**Supplemental Figure 5. Tumor-specific mortality of desloratadine users vs non-users with gastric cancer.** Gastric cancer-specific survival probability plotted against time since diagnosis in years.**
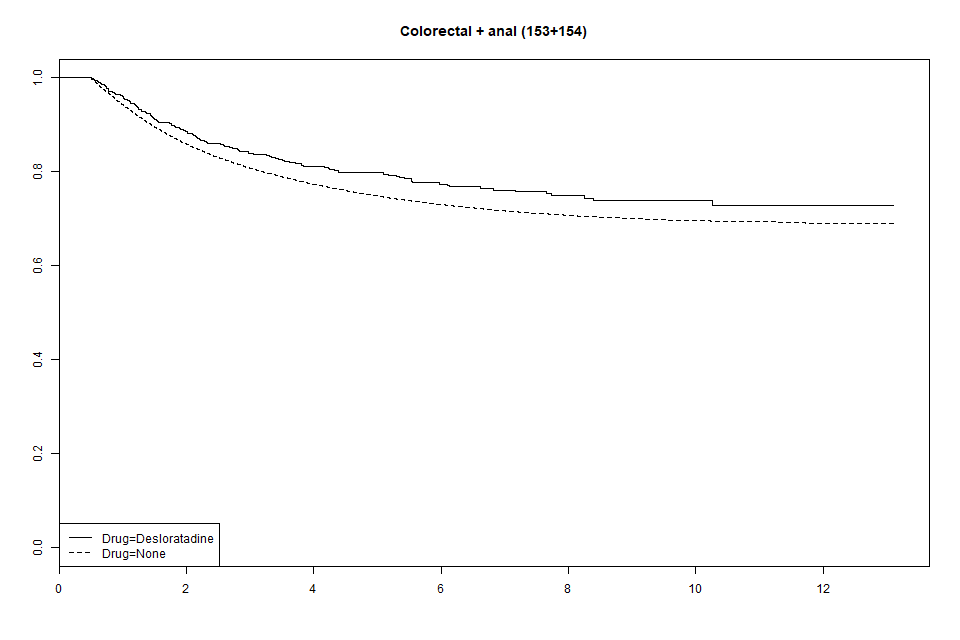
Supplemental Figure 6. Tumor-specific mortality of desloratadine users vs non-users with colorectal/anal cancer.** Colorectal/anal cancer-specific survival probability plotted against time since diagnosis in years.

**
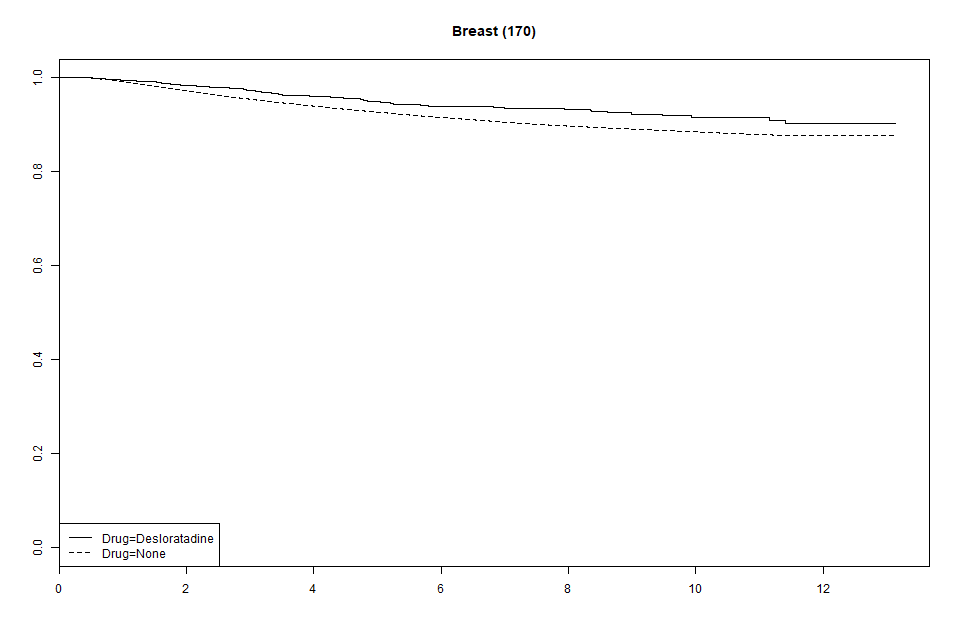
**

**Supplemental Figure 7. Tumor-specific mortality of desloratadine users vs non-users with breast cancer.** Breast cancer-specific survival probability plotted against time since diagnosis in years.
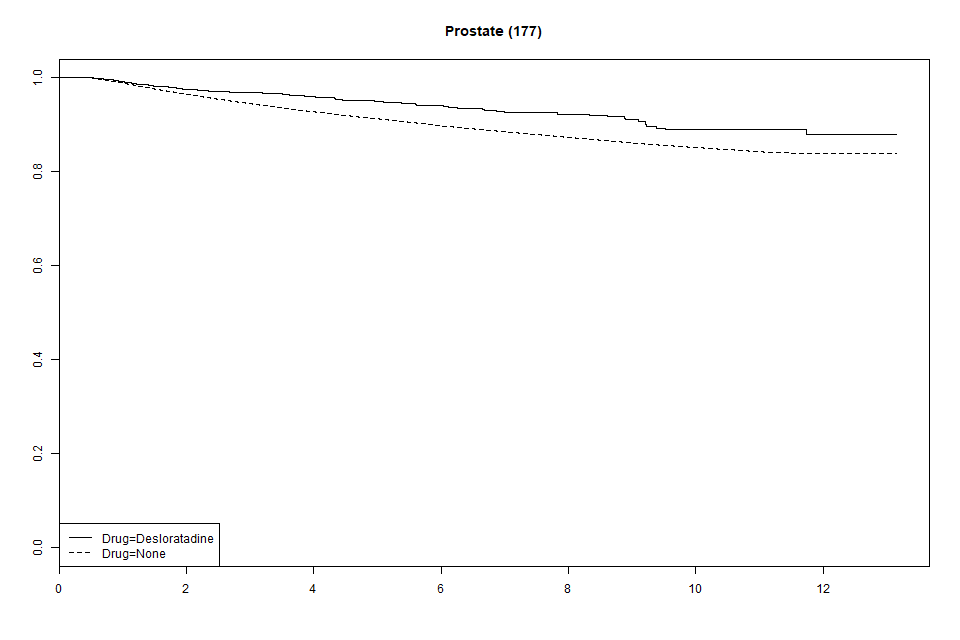


**Supplemental Figure 8. Tumor-specific mortality of desloratadine users vs non-users with prostate cancer.** Prostate cancer-specific survival probability plotted against time since diagnosis in years.

**
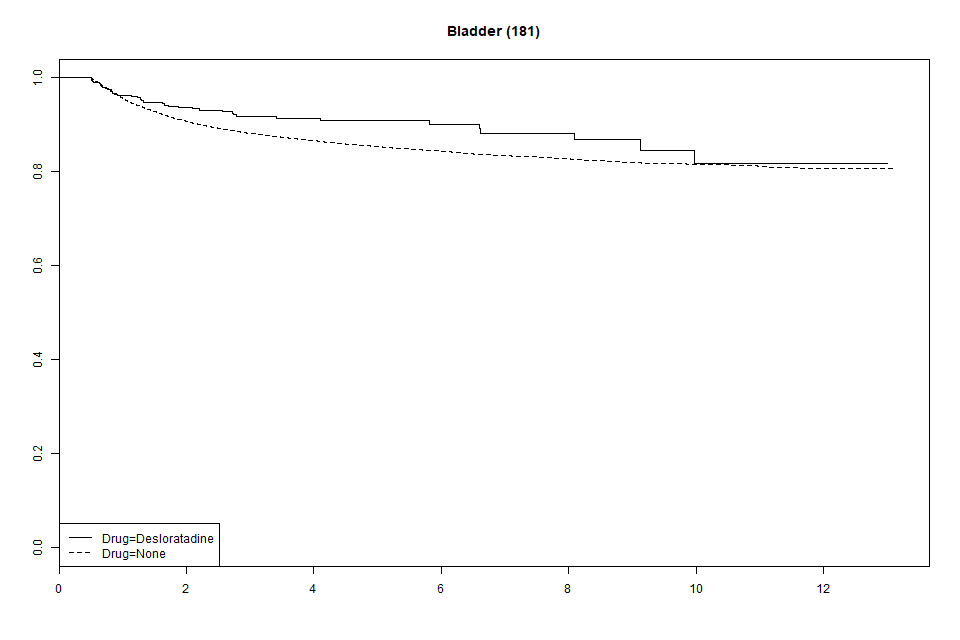
Supplemental Figure 9. Tumor-specific mortality of desloratadine users vs non-users with bladder cancer.** Bladder cancer-specific survival probability plotted against time since diagnosis in years.

**
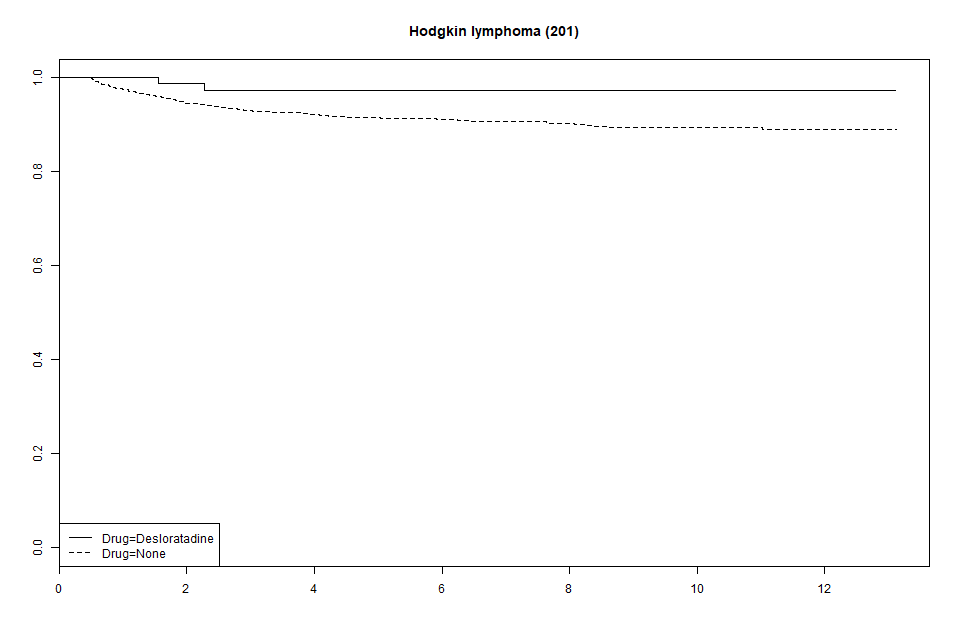
Supplemental Figure 10. Tumor-specific mortality of desloratadine users vs non-users with Hodgkin lymphoma.** Hodgkin lymphoma-specific survival probability plotted against time since diagnosis in years.
